# Prevalence, aetiology, treatment and outcomes of shock in children admitted to Kenyan hospitals

**DOI:** 10.1186/s12916-016-0728-x

**Published:** 2016-11-16

**Authors:** George Mbevi, Philip Ayieko, Grace Irimu, Samuel Akech, Mike English, on behalf of the Clinical Information Network authors, Samuel Ng’arng’ar, Nick Aduro, Loice Mutai, David Kimutai, Caren Emadau, Cecilia Mutiso, Celia Muturi, Charles Nzioki, Francis Kanyingi, Agnes Mithamo, Magdalene Kuria, Sam Otido, Anne Kamunya, Alice Kariuki, Peris Njiiri, Rachel Inginia, Melab Musabi, Barnabas Kigen, Grace Akech, Lydia Thuranira, Morris Ogero, Thomas Julius, Boniface Makone, Mercy Chepkirui, Wycliffe Nyachiro, James Wafula

**Affiliations:** 1 Kenya Medical Research Institute/Wellcome Trust Research Programme, P.O. Box 43640-00100, Nairobi, Kenya; 2 Department of Paediatrics and Child Health, University of Nairobi, Nairobi, Kenya; 3 Nuffield Department of Medicine, Oxford University, Oxford, UK

**Keywords:** Shock, Children, Prevalence, Bolus, Kenya

## Abstract

**Background:**

Shock may complicate several acute childhood illnesses in hospitals within low-income countries and has a high case fatality. Hypovolemic shock secondary to diarrhoea/dehydration and septic shock are thought to be common, but there are few reliable data on prevalence or treatment that differ for the two major forms of shock. Examining prevalence and treatment practices has become important since reports suggest high risks from liberal use of fluid boluses in African children. The present study aims to estimate the prevalence, fluid management practices and outcomes of shock among hospitalised children.

**Methods:**

We analysed paediatric in-patient data collected using discharge case record review between October 2013 and February 2016 from 14 hospitals in Kenya which are part of a network (referred to as the Clinical Information Network) using similar tools for standardised clinical records with care directed by the local clinical team leaders. Data are from a period after dissemination of national guidance seeking to limit use of bolus fluids.

**Results:**

A total of 74,402 children were admitted between October 2013 and February 2016. Children aged < 30 days or > 5 years, with severe acute malnutrition, surgical/burns, or cases with pre-defined minimum data sets were excluded from analysis. This resulted in 42,937 patients meeting the inclusion criteria. Prevalence of clinically diagnosed shock was 1.5 % (n = 622) and overall bolus use was 0.9 % (n = 366); 41 % (256/622) of children with clinically diagnosed shock did not receive a fluid bolus (but had a fluid plan for management of dehydration). Identified cases appeared mostly to be hypovolaemic shock secondary to dehydration/diarrhoea (94 %, 582/622), with a high case fatality (34 %, 211/622). Overall mortality for all admitted children was 5 % (2115/42,937) and was 7.9 % (798/10,096) in children with dehydration/diarrhoea. The diagnosis of hypovolaemic shock was nearly always accompanied by additional clinical diagnosis (99 %), most often pneumonia or malaria. Where bolus fluids were used, they were prescribed in accordance with guidelines (isotonic fluid at correct volume) in 92 % of cases. Inappropriate use of bolus fluids to treat milder forms of impaired circulation appeared very rarely.

**Conclusion:**

A diagnosis of shock is uncommon at admission and use of fluid bolus is rare in admissions to Kenyan hospitals. A fluid bolus, when prescribed, is mostly used in children with hypovolemic shock secondary to dehydration and case fatality in these cases is high. We found little evidence of liberal use of fluid bolus that might cause harm in a period following dissemination of national guidelines suggesting very strict criteria for fluid bolus use.

## Background

Shock, defined as relative or absolute reduction of circulatory volume with consequent impaired tissue perfusion, oxygen delivery and waste removal, has a high risk of mortality and requires prompt appropriate intervention [1, 2]. However, there is no consistent clinical definition of shock and it may have a number of underlying causes, including cardiac, sepsis, hypovolaemia and anaphylaxis [3–7]. Sepsis is thought to be the most common cause of shock in high-income countries, but hypovolaemia secondary to diarrhoea/dehydration is thought to be predominant in low-income countries (LICs). The World Health Organization (WHO) does not formally recognise shock in its diarrhoea/dehydration case management guidelines [8], but this is provided for in its Emergency Triage, Assessment and Treatment (ETAT) guidelines [9]. However, the prevalence of shock (hypovolemic or septic) is poorly described in LICs [10, 11]. In the FEAST trial, a large trial of fluid bolus resuscitation, up to 57 % of acutely ill, febrile admitted children had features associated with impaired perfusion, but only 2 % of participants enrolled met ETAT criteria for shock [12]. The FEAST trial and the debate that followed its publication showed that there’s little understanding on how commonly clinicians diagnose shock in routine settings, what they associate the diagnosis with, and how often they initiate treatment for shock with fluid bolus. The latter is of particular significance as bolus fluids were shown to be harmful in the FEAST trial when used in children without diarrhoea/dehydration [7, 13]. A liberal approach to use of fluid bolus might therefore be causing considerable harm in hospitalised children in LICs where intensive care cannot be provided. This analysis aims to address these gaps in understanding by exploring the prevalence of shock, its clinically determined aetiology, fluid management practices and outcomes among hospitalised children aged under 5 years. It will also help to estimate the magnitude of potential harm if shock is treated with fluid boluses in non-diarrhoeal cases. The study uses routine data collected from a network of 14 hospitals in Kenya referred to as the Clinical Information Network (CIN).

## Methods

### Study setting and data collection

Data for analysis was routinely obtained from the 14 hospitals in Kenya that form the CIN, which is a collaborative effort between the Kenya Medical Research Institute (KEMRI), the Kenya Paediatric Association, and Kenya’s Ministry of Health that aims to improve the availability of data for decision making and to help improve quality of care. A detailed description of the CIN hospitals, their selection process, management and geographical spread has been previously provided [14]. In summary, key partners at each site consist of the hospital paediatrician, the nurse in charge of the paediatric unit and the senior health records officer. A minimum dataset, which consists of information required for national reporting of basic demographic features, diagnoses and outcomes only, is collected for each admission in all hospitals. A more comprehensive set of information on disease care processes, which includes investigations and treatment, is collected on all patients in 11 hospitals with low-to-moderate workload and on a random subset in three high workload hospitals. Minimum datasets are also collected when the data clerk is on leave, for surgical/burns cases (because they are primarily admitted outside paediatric areas), and on neonates admitted to the paediatric wards. Minimum data sets do not have detailed treatment information and were therefore excluded. Partnership is maintained through 4-monthly meetings, 2-monthly written reports to hospitals and phone calls by the network coordinator to the hospitals. The hospitals use a standardised paediatrics admission record and discharge forms and have one additional records clerk to collect data from medical records and laboratory reports. A sample of the paediatrics admission record form can be found at the iDOC Africa website [15]. Data is extracted from these forms as soon as a patient is discharged and entered into a non-proprietary electronic tool, REDCap® [16], in line with detailed standard operating procedures. Data on clinical presentation, assessment, care processes for common conditions, investigations and treatment given within the first 48 hours are collected together with discharge diagnoses and outcomes. The database tool has been configured to run error checks so corrections can be made on site before the data are uploaded and synchronised into a central server. Further quality checks are done once the data are synchronised and the clerks alerted to reconcile any discrepancies noted. Data quality assurance, where the study team randomly selects a sample of files that have been entered by the clerks and re-enters the same data, is done periodically to ascertain the accuracy of data from the hospitals and as a form of supervision. WHO syndromic criteria [8] are used to assign diagnoses for common clinical conditions by clinicians, in line with national guidelines, with ICD-10 also entered for each case. WHO syndromic definitions are used as diagnostic categories in this paper. A detailed description of data collection is provided elsewhere [17]. Data collection was commenced between October 2013 and February 2014 in the 14 hospitals.

### Data analysis

Data collection from the initiation of the network up to February 2016 were analysed. Children with severe acute malnutrition, aged < 30 days, with surgical/burns, those with minimal data sets, and those aged > 5 years were excluded. Severe malnutrition cases were excluded as they have distinct management fluid guidelines. Neonates (<30 days old) were excluded because there is lack of consensus on fluid management guidance with no clear policy in Kenya, while children > 5 years old are not covered by the Kenyan guidance that is the main focus of the CIN. Otherwise, all cases with comprehensive data not meeting exclusion criteria were included in the analyses. To examine the prevalence of shock amongst admissions, shock was defined as a child with any of the following: a clinician’s indication that the child had shock as a problem accompanying diarrhoea and dehydration (an indication of the severity of fluid loss); a diagnosis of shock associated with an underlying cause (e.g. septic shock); or use of rapid bolus fluid therapy in a child irrespective of diagnosis. When attributing a probable cause for an admission with shock (as defined above) we considered it to be hypovolaemic (hypovolaemic shock) in nature in children with a diagnosis of diarrhoea and dehydration and non-hypovolaemic (other) in the absence of this diagnosis. We examined the fluids prescribed for the treatment of shock and calculated the volume per kilogram body weight based on the fluid therapy plan and the recorded admission weight. We also examined the recording of key clinical features used in guidelines to support the diagnosis of shock. Children fulfilled locally adapted WHO criteria for shock (documented in national clinical policy since 2013) if they had all four of the following: impaired consciousness (AVPU score < V), weak/absent pulse, cold hands and temperature gradient, and capillary refill > 3 seconds, plus sunken eyes and slow skin pinch. The presence of shock plus diarrhoea symptoms classified such cases as diarrhoea-related hypovolaemic shock. Impaired circulation was defined as presence of any one of weak/absent pulse, cold hands and temperature gradient, or capillary refill > 3 seconds. Information on prevalence is summarised as proportions with shock or impaired circulation among those admitted. Fluid treatment of shock, number of boluses, types of shock noted, and case fatality are summarised as frequencies and proportions. Children with diarrhoea/dehydration and shock were defined to have comorbidity if there was another clinical diagnosis in addition to diarrhoea/dehydration. No imputation for missing data was done and because this affects denominators, all numerators and denominators are reported.

## Results

### Prevalence of shock, patient characteristics, and use of fluid bolus

**Fig. 1:**
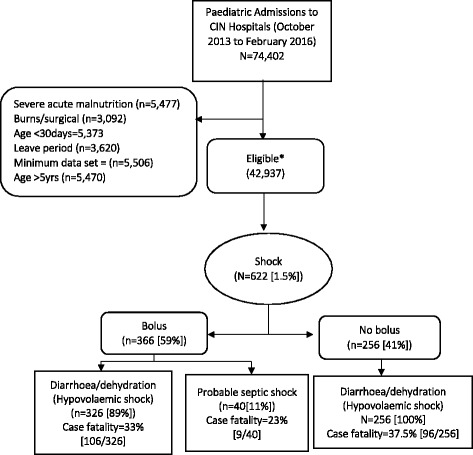
Flow diagram showing patients with shock and bolus administration. * 41 (0.1%) children met WHO criteria for shock and 7.5% (3,219/42,937) had impaired circulation; 11% (366/3,219) of children with impaired circulation received fluid bolus

A total 74,402 children were admitted between October 2013 to February 2016 and 42,937 records (range 1154 to 5924 per hospital) were analysed after applying the exclusion criteria above. Characteristics for the 42,937 participants are summarised in Table 1. Shock (as defined in the Methods section) was an uncommon diagnosis and was only present in 1.45 % (622) of admitted children (range 0.2–3.2 % per hospital); in 1.4 % (582/622) of all admissions, the shock appeared to result from hypovolaemia secondary to diarrhoea/dehydration, while in 0.1 % of all admissions (40/622) shock was identified in non-diarrhoeal cases (other shock). Sixty percent (24/40) of those with other shock had fever and septic shock was a possibility. Therefore, hypovolaemic shock comprised 94 % (582/622) of all cases of shock diagnosed. A summary of shock diagnosis and fluid bolus use is presented in Fig. 1. WHO criteria for shock were fulfilled in only 0.1 % (41/42,937) of children. When individual clinical signs were considered, 7.5 % (3219/42,937) of children in the study population met criteria for impaired circulation; of these, 11 % (366/3219) of children with impaired circulation received rapid fluid bolus. Forty three percent (265/622) of patients with shock were female, 33 % (204/622) had malaria, 1.4 % (9/622) had HIV, 46 % (286/622) had pneumonia, 1.1 % (7/622) had tuberculosis, 13 % (81/622) had meningitis, 1.1 % (7/622) had asthma, and 2.9 % (18/622) had rickets (Table 2).

**Table 1:** Description of characteristics of eligible population

Overall characteristics			
Demographic characteristics	n (%)		
Female sex, n (%)^a^	18,753 (43.7)		
Anthropometric measures	Mean (± SD)		
Age in months	15.0 (11.7)		
Weight, kg	8.7 (2.8)		
Weight–height Z score	–1.3 (1.2)		
MUAC^b^, cm	13.5 (1.6)		
Height/length, cm	74.3 (11.4)		
Illness features	Mean (± SD)		
Length of illness, days	4.3 (4.7)		
Oxygen saturation	94.9 (6.2)		
Heart rate/minute	124.2 (27.9)		
Respiratory rate/minute	41.8 (13.4)		
Axillary temperature (°C)	37.7 (1.2)		
Airway and breathing	Characteristic present, n (%)	Characteristics absent, n (%)	Missing, n (%)
Stridor	783 (1.8)	34,300 (79.9)	7854 (18.3)
Grunting	4165 (9.7)	32,192 (75.0)	6580 (15.3)
Crackles	8425 (19.6)	29,004 (67.6)	5508 (12.8)
Indrawing	11,383 (26.5)	25,697 (59.8)	5857 (13.6)
Tachypnoea^c^	13,236 (30.8)	18,240 (42.5)	11,461 (26.7)
Acidotic breathing	1019 (2.4)	34,935 (81.4)	6983 (16.3)
Central cyanosis	253 (0.6)	38,266 (89.1)	4418 (10.3)
Circulation			
Tachycardia^d^	10,278 (23.9)	14,643 (34.1)	18,016 (42.0)
Sunken eyes	4749 (11.1)	29,158 (67.9)	9030 (21.0)
Delayed skin pinch	6702 (15.6)	28,077 (65.4)	8158 (19.0)
Capillary refill > 2 seconds	1620 (3.8)	27,352 (63.7)	13,965 (32.5)
Capillary refill > 3 seconds	312 (0.7)	28,660 (66.7)	13,965 (32.5)
Temperature gradient^e^	1432 (3.3)	27,313 (63.6)	14,192 (33.1)
Weak pulse volume	1766 (4.1)	32,951 (76.7)	8220 (19.1)
Pallor	5962 (13.9)	32,403 (75.5)	4572 (10.6)
Disability			
Convulsions	9228 (21.5)	28,613 (66.6)	5096 (11.9)
Can drink/breastfeed?	29,586 (68.9)	5757 (13.4)	7594 (17.7)
Stiff neck	871 (2.0)	36,645 (85.3)	5421 (12.6)
Bulging fontanelle	453 (1.1)	34,861 (81.2)	7623 (17.8)
Impaired consciousness^f^	2464 (5.7)	35,485 (82.6)	4988 (11.6)
Diagnoses			
Anaemia	3774 (8.8)	39,163 (91.2)	Nil
Asthma	1262 (2.9)	41,675 (97.1)	Nil
Dehydration	7021 (16.4)	35,916 (83.6)	Nil
HIV	401 (0.9)	355 (0.8)	42,181 (98.2)
Malaria	17,254 (40.2)	25,683 (59.8)	Nil
Meningitis	5393 (12.6)	37,544 (87.4)	Nil
Pneumonia	20,038 (46.7)	22,899 (53.3)	Nil
Ricketts	962 (2.2)	41,975 (97.8)	Nil
Tuberculosis	545 (1.3)	42,392 (98.7)	Nil
Outcome			
Death	2115 (4.9 %)	n/a	Nil

Table describes characteristics of all 42,937 patients who were eligible for analysis; means presented rather than median because data were not skewed

^a^489 (1.1 %) patients missed information on sex

^b^Mid-upper arm circumference

^c^Defined as > 50 breaths/minute if aged ≤ 1 year and > 40 breaths/minute if aged > 1 year

^d^Defined as > 140 beats/minute if aged ≤ 1 year and > 120 beats/minute if aged > 1 year

^e^Temperature gradient

^f^AVPU score < A

**Table 2:** Clinical diagnoses of patients with shock admitted to Clinical Information Network hospitals

	Hypovolaemic shock treated with bolus (n = 326) n (%)	Hypovolaemic shock not given fluid bolus (n = 256) n (%)	Other shock (all received bolus) (n = 40) n (%)	All shock (n = 622) n (%)
Malaria^a^	106 (33)	80 (31)	18 (45)	204 (33)
Pneumonia^a^	135 (41)	132 (52)	19 (48)	285 (46)
HIV positive or exposed^a^	5 (1.5)	4 (1.5)	0	9 (1.4)
Tuberculosis^a^	2 (0.6)	4 (1.6)	1 (2.5)	7 (1.1)
Meningitis^a^	26 (8)	50 (20)	5 (13)	81 (13)
Asthma^a^	2 (0.6)	5 (2)	0	7 (1.1)
Rickets^a^	5 (1.5)	13 (5)	0	18 (2.9)

^a^Condition is the presenting comorbidity in cases of hypovolaemic shock

Fluid bolus was prescribed in only 0.85 % (366/42,937) of all admissions. Most boluses (89 %, 326/366) were given for hypovolaemic shock and only 11 % (40/366) were given for non-diarrhoeal shock (other shock). Forty four percent (256/582) of children with diarrhoea/dehydration identified as having shock (hypovolaemia) did not receive rapid fluid bolus but received recommended fluid management of dehydration (WHO plan C). All the children with shock who did not receive a rapid fluid bolus (but other recommended fluid management) had hypovolaemia.

When a fluid bolus was prescribed, it was defined as correctly prescribed if an isotonic fluid (normal saline or Ringer’s lactate/Hartmann’s solution) was used at a volume of 20 mL/kg (±4 mL/kg) and was correct in 92 % of cases. In a small number of cases (n = 30, 8 %), fluids such as Half Strength Darrow’s/5 % Dextrose mix, Ringer’s/5 % Dextrose mix, or 5 % Dextrose were used for fluid boluses. One bolus was infused in 88 % of recipients, 10 % received two boluses (all except one were in children with diarrhoea/dehydration), and only one patient each received 3 and 4 boluses (these children did not have diarrhoea; one had severe anaemia/suspected sepsis and the other had suspected sepsis).

### Mortality and comorbidities

Five percent of all admission (n = 2115/42,937) died and 10 % (211/2115) of these deaths were associated with clinically diagnosed shock. Mortality in children identified with shock was 34 % (211/622), and mortality was 31 % (115/366) in the rapid bolus subgroup and 37.5 % (96/256) in the no bolus subgroup (odds ratio (OR) = 0.76; 95 % confidence interval (CI), 0.55–1.07; *P* = 0.12). Among children with hypovolaemic shock, mortality in children who received rapid fluid bolus was 32.5 % (106/326) compared to 37.5 % (96/256) in those who only received WHO plan C (OR = 0.80; 95 % CI, 0.57–1.13; *P* = 0.21).

Most children with hypovolaemic shock had a second diagnosis (referred to as comorbidity) and this was seen in 99 % (579/582) of cases. Additional diagnoses in patients with shock are outlined in Table 2. Patients with hypovolaemic shock and a comorbid condition had an increased risk of death when compared to those with diarrhoea only (109/300 (36 %) vs. 35/147 (24 %), respectively; OR = 1.9; 95 % CI, 1.2–2.9; *P* = 0.008). Mortality in those with hypovolaemia plus malaria was 29 % (54/186) and in those with hypovolemia plus pneumonia it was 42 % (111/267).

## Discussion

We analysed data from CIN hospitals for prevalence of shock and use of bolus fluids in order to give an estimation of the prevalence of shock at admission and the use of rapid fluid bolus. The study was undertaken in the period immediately after dissemination of national guidelines suggesting rapid bolus fluids should only be used in children with four clinical features indicating shock who also had a history of diarrhoea and two signs of dehydration (sunken eyes and prolonged skin pinch). The data suggest shock is uncommonly diagnosed at admission in children aged 1 month to 59 months and that most cases are diagnosed in the context of multiple signs of hypovolaemia secondary to diarrhoea/dehydration. Thus, while just under 1.5 % of admissions were diagnosed with shock, approximately 7.5 % of admissions were noted to have at least one of the four clinical signs associated with impaired perfusion. Use of fluid bolus is rare (<1 % admissions) and is mostly used in those children with clinically identified hypovolaemia and dehydration (most with hypovolaemic shock).

Although shock is identified rarely, it has a high case fatality and 10 % of all deaths were associated with the clinical syndrome. Nearly all cases of shock secondary to diarrhoea/dehydration also had a second diagnosis (comorbidities) and having an additional diagnosis was associated with an increased risk of death. Clinicians mostly prescribed the guidance recommended bolus type and volumes [18] and in 9/10 cases only a single bolus is given although many children with diarrhoea/dehydration will then go on to continue with intravenous fluids as part of ongoing management. The use of correctly calculated fluid boluses may be attributed to long term efforts to provide training in emergency care in Kenya using the ETAT+ course and linked efforts to disseminate evidence-based national guidelines that provide this information [19, 20]. However, there remains uncertainty about the benefits of rapid bolus fluids even in children with all four clinical signs of shock, a history of diarrhoea and signs of dehydration. With a fatality rate of over 30 % in this group, further studies of the benefits and risks of aggressive fluid intervention may be warranted.

These data from 14 Kenyan hospitals suggest that clinicians rarely use fluid bolus to treat non-hypovolaemic shock and extremely rarely use such boluses in those with signs of impaired circulation only. As the FEAST trial showed increased risk of mortality in children with shock (defined more broadly than in this study) from non-diarrhoeal cases and those who only have impaired circulation, these data are therefore reassuring and perhaps allay fears that considerable harm is being done through widespread use of bolus fluids [12]. These data do, however, represent a period in Kenya immediately after a change in national guidelines, disseminated in October 2013, recommending against use of fast-bolus infusions in the absence of diarrhoea/dehydration. Such Kenyan guidance was based on systematic review [21] and a structured national guideline panel process [22]. Kenyan guidance predated the advice of WHO that also now recommends against fast fluid bolus in the absence of diarrhoea/dehydration in its ETAT guidance released in 2016 [3, 9]. This new WHO guidance recommends (based on expert opinion) early consideration of inotropes, antibiotics and other treatments in the septic shock management algorithm for cases that fail to respond to initial, more cautious fluid bolus. This continued recommendation for some form of fluid bolus remains contentious. Our finding that most children even with hypovolaemic shock associated with diarrhoea/dehydration also have a secondary diagnosis of infection (predominantly malaria and pneumonia) for which rapid fluid boluses are thought to be harmful brings into question how frontline clinicians, without expert training in critical care and with little access to diagnostics, are expected to apply divergent guidelines to manage a single patient.

There have been concerns that there is a high prevalence of severe sepsis in LICs with higher case fatality than in high-income countries [10, 11]. High prevalence of sepsis in LICs might be expected to correspond with a common diagnosis of shock associated with febrile illness but not associated diarrhoea/dehydration. Our data suggest clinicians are not often making a diagnosis at admission of probable septic shock (non-diarrhoeal shock), suggesting that fulminant septic shock may still be rare in Kenya. However, the diagnoses we report are almost exclusively made on the basis of clinical signs interpreted often by clinicians with limited experience. Typically, measures of blood pressure and oxygen saturation are not available and neither are microbiological investigations of those for inflammatory markers [23]. Furthermore, although we had access to discharge diagnoses, it is possible that shock emerged during the admission but these diagnoses were not recorded. Our estimates of prevalence of non-hypovolaemic shock may therefore be low. In addition, the clinical identification of shock and impaired circulation in Kenyan guidelines does not include children with severe tachycardia, a criterion used in the FEAST study. Our study also included children with diarrhoea/dehydration, many of whom were excluded from the FEAS study. Such differences in definitions make direct comparison with the prevalence of impaired circulation and of mortality between the two reports difficult. However, we believe CIN hospitals are fairly representative of practice in Kenyan hospitals in LICs providing first referral level care, although the fact that they receive regular feedback on their patterns of mortality and morbidity and are engaged in a network aiming to improve their documentation of illness over time [14] may result in better adherence to recently disseminated national guidelines for fluid management in keeping with previous efforts to use multifaceted interventions, including feedback, to improve adherence to guidelines in Kenya [24–26].

These findings need to be interpreted with consideration for known limitations pertinent in registry research such as ascertainment, selection and analytical biases [27]. However, we suggest selection bias was unlikely because we analysed all eligible children across all 14 hospitals. Ascertainment bias was reduced by using WHO standardised case definitions, standard data collection approaches and employing frequent data quality assurance. However, results are based on what is recorded about the care provided and it is possible that bolus fluids were used but not recorded or that they were prescribed but not given.

Improved use of simple bedside physiological measures together with biomarkers might improve the diagnosis of shock, help subclassify the clinical presentation by cause and assist in management including targeted anti-microbial or inotropic use. While fewer than 10 % of the deaths amongst the cohort studied could be directly associated with a diagnosis of shock on admission, in these cases, case fatality was very high and some cases may go unidentified. Comorbidity further increases the risk of mortality in hypovolaemic shock. This constellation of results suggests that the saving of lives associated with shock will likely require detection and intervention earlier in the disease course, more rapid referral where needed and improved supportive care. It is also possible that different treatment approaches are needed in children with different patterns of comorbidity. For example, less aggressive fluid regimens might perhaps be tested, particularly in children with hypovolaemic shock and signs of pneumonia. Such research will require large scale collaborative efforts that could benefit from initiatives such as the clinical information network in Kenya.

## Conclusion

The diagnosis of shock is uncommon at admission and use of fluid bolus is rare. Fluid bolus is mostly used in children with hypovolemic shock secondary to dehydration and clinicians mostly prescribe the fluid and amounts recommended in current guidance. Further improvements can be made in identifying and treating children with shock, perhaps particularly to improve availability of simple bedside monitoring devices.
